# Assessing the gap between policy and practice: community health workers’ contributions to nutrition services in Sub-Saharan Africa

**DOI:** 10.1186/s12913-025-13044-6

**Published:** 2025-07-01

**Authors:** Akriti Singh, Mahamadou Mansour Ndiath, Djeinam Toure, Romance Dissieka, Lennie Kyomuhangi Bazira, Carolyne Wanyonyi, Rolf DW Klemm

**Affiliations:** 1 Helen Keller Intl, Dakar, Africa Region Senegal; 2 Helen Keller Intl, New York, NY USA; 3 Community Health Impact Coalition, Clarksville, MD USA; 4 Community Health Impact Coalition, Nairobi, Kenya; 5 Johns Hopkins Bloomberg School of Public Health, Baltimore, MD USA

**Keywords:** Maternal health, Child health, Nutrition services, Community health workers, Sub-Saharan Africa, Policy analysis

## Abstract

**Background:**

Community health workers (CHWs) are the backbone of healthcare service provision in Africa, particularly in delivering critical health and nutrition services. Despite their pivotal role, there is limited understanding of the alignment between CHW responsibilities as defined in national policies and the actual nutrition services they provide. This study aimed to compare the nutrition-related responsibilities assigned to CHWs in national policies with the nutrition services CHWs report delivering to women and children in six Sub-Saharan African countries: Guinea, Mali, Niger, Côte d’Ivoire, Democratic Republic of Congo (DRC), and Kenya.

**Methods:**

We combined structured interviews with 735 CHWs from six countries with a document review of national CHW policies and training manuals to assess the alignment between policy and practice in nutrition service delivery.

**Results:**

The policy documents revealed notable differences in the CHW scope of responsibilities across the six countries. Training for CHWs was inconsistently documented across countries. Financial compensation for CHWs was mentioned in policy documents from five out of six countries, though the nature and amount of compensation varied significantly. Among the CHWs surveyed, a high percentage (68-84%) reported receiving financial compensation. Most CHWs provided promotional services but were more likely to dispense preventive and curative commodities for children (e.g., Vitamin A, deworming, oral rehydration solution, and Zinc) than women (iron-folic acid, intermittent preventive treatment for Malaria). Service provision was closely linked to the training received. Discrepancies were noted between policy-defined responsibilities and reported service delivery, particularly in growth monitoring and promotion and management of wasting.

**Conclusions:**

This study found a critical need for stronger alignment between policy directives and CHW training and service provision. By informing policy reforms, standardizing training, and guiding resource allocation, our findings can strengthen CHW programs and improve delivery of lifesaving nutrition interventions to women and children—particularly in remote and underserved communities.

**Supplementary Information:**

The online version contains supplementary material available at 10.1186/s12913-025-13044-6.

## Background

The World Health Organization (WHO) estimates that by 2030 there will be a global shortage of 10 million health workers [1]. To address this gap, WHO and several others have encouraged countries to adopt a mix of approaches to provide care, including the potential of community health workers (CHWs) [1, 2]. CHWs are the backbone of healthcare service provision in Africa and in 2017 the African Union committed to supporting 2 million CHWs. As trusted members of their communities, CHWs facilitate community engagement, build trust, and ensure that health and nutrition interventions are culturally sensitive and well-received [3]. CHWs deliver critical primary health care services such as immunization, nutrition supplementation, and maternal and child health care [4]. They often play a vital role in ensuring that these services are accessible, especially in rural areas where distances to health facilities are long [5]. They also provide education on various health and nutrition topics, empowering community members with knowledge and skills to improve their health and well-being [4].

Among the services CHWs deliver to their communities, nutrition doesn’t always get the attention it deserves. Nutrition is a cornerstone of maternal and child health, and survival, and inadequate nutrition can have long-lasting effects on growth, development, and overall well-being. CHWs are uniquely positioned to address maternal and child nutrition-related challenges within their communities by delivering promotional and curative services. Evidence shows that CHWs can successfully deliver maternal nutrition services leading to an uptake of micronutrient supplements (e.g., micronutrient supplements, iron-folic acid (IFA), calcium), improved breastfeeding and complementary feeding practices, and better diet quality [6]. CHWs can also effectively deliver child nutrition services such sick childcare; distributing essential micronutrients (e.g., Vitamin A and multiple micronutrient powders); and managing cases of wasting [7–13]. Despite their significant contribution to advancing nutrition in their communities, several papers have highlighted training and compensation as barriers to quality nutrition service delivery by CHWs [11, 14]. National policies typically define the nutrition-related responsibilities of CHWs as well as their training and compensation strategy. However, limited studies have examined the alignment between national policies and actual service delivery.

This study seeks to fill this gap by comparing the nutrition-related responsibilities assigned to CHWs in national policies with the services they report delivering across six countries. Understanding this alignment—or lack thereof—is critical for several reasons. First, discrepancies between policy and practice can undermine the effectiveness of CHWs, leading to suboptimal health outcomes. Second, by identifying gaps in training, compensation, and service provision, policymakers and program designers can better support CHWs, thereby enhancing their capacity to deliver lifesaving nutrition interventions. Finally, this study aims to contribute to the broader discourse on strengthening health systems in Sub-Saharan Africa, where CHWs are often the first and sometimes the only point of contact for healthcare in many communities.

Specifically, this study is guided by the following research questions:

1. To what extent do national policies in Côte d’Ivoire; Democratic Republic of Congo (DRC); Guinea; Mali; Niger; and Kenya define the nutrition-related responsibilities of CHWs?
2. What nutrition services do CHWs report delivering to women and children under five years of age in these countries?
3. How does the reported service provision align with the responsibilities outlined in national policies?
4. What are the key enablers and barriers influencing CHWs’ ability to deliver these nutrition services, particularly regarding training and financial compensation?

## Methods

This study employed a mixed-methods design combining quantitative survey data with a structured document review to address our four research questions. To assess community health workers’ (CHWs) service delivery, we analyzed secondary data collected through post-event coverage (PEC) surveys for Vitamin A Supplementation (VAS). To understand how national policy frameworks define CHW roles, we conducted a review of official policy documents and training materials. Finally, to explore gaps and alignment between policy and practice, we compared findings from both data sources. These complementary methods enabled us to examine not only what services CHWs provide, but also how those responsibilities are formally articulated in national guidance.

### CHW survey

#### Study design and sampling

Data from community health workers were collected as part of the post-event coverage (PEC) surveys for Vitamin A supplementation (VAS). The survey utilized a cross-sectional design, with data collected through structured interviews with community health workers (CHWs) across six Sub-Saharan African countries: Côte d’Ivoire, Democratic Republic of Congo (DRC), Guinea, Mali, Niger, and Kenya. These surveys assess the reach and effectiveness of Vitamin A distribution using a two-stage random cluster design with probability proportional to size (PPS) sampling.

In the first stage, we defined clusters or enumeration areas (EAs), which we randomly chose using the PPS method from lists provided by each country’s national statistics bureau. This approach ensured that areas with larger populations had a higher probability of being selected, while also preserving proportional representation across geographic regions and urban–rural strata. The number of clusters selected in each country reflected both the population size and geographic dispersion within each VAS coverage region to ensure broad inclusion.

In the second stage, one health facility was randomly selected within each chosen cluster. Because most health facilities serve a defined population within the EA or immediate catchment area, this approach allowed us to randomly access the community health system within the sampled geographic area. By then randomly selecting a CHW affiliated with that facility—who had participated in the most recent VAS campaign—we ensured that the CHWs interviewed were actively engaged in relevant service delivery in the sampled area, thus preserving internal consistency and practical representativeness within each selected cluster. This sampling design, while not exhaustive, was intended to yield a representative snapshot of CHW service delivery within each VAS coverage region. Additional details on the sampling frames and regions selected are available in previously published work [15].

Some countries have two groups of community-level service providers. The community health workers we sampled belonged to the following cadre: community health agents (Côte d’Ivoire); community liaisons (Democratic Republic of Congo), community liaisons (Guinea); community health workers (Mali); community liaisons (Niger); community health promoters (Kenya). The surveys with community health workers were conducted between June and December 2023. After data collection for the survey had completed, we held a validation workshop to present findings and gather feedback from stakeholders including representatives from the Ministry of Health, regional and district health offices, and health facilities to ensure accuracy and credibility of findings.

#### Informed consent

Informed consent was obtained from all participating CHWs before the interviews. The consent process was tailored to the cultural contexts of each country, ensuring that participants fully understood the study’s purpose, the voluntary nature of their participation, and their right to withdraw at any time without penalty. Written consent forms were provided in the local languages, and where necessary, verbal consent was obtained and documented, particularly in settings with low literacy levels. The consent process was facilitated by trained interviewers who explained the study objectives, the confidentiality of the data, and how the findings would be used to inform policy and practice in their respective countries.

### Document review

We retrieved national community health policies from a publicly available policy dashboard [16]. For additional documents such as specific training materials, we contacted staff from the authors’ institution office in the six countries, as needed.

### Data analysis

We used Stata version 17 for analysis (StataCorp, 2021). We conducted bivariate analyses to compare the provision of services in communities and training among CHWs across the six countries included in the study. The services analyzed for women included iron and folic acid (IFA) and intermittent preventive therapy (IPT) during pregnancy and infant and young child feeding (IYCF) counseling. For children, we examined growth monitoring and promotion (GMP), management of wasting, vitamin A supplementation (VAS), deworming, and diarrhea management. For each of these services, we reported the percentage of CHWs who indicated that they provided the service, the frequency with which the service was provided where relevant, and the percentage of CHWs who reported receiving training specific to each service.

To contextualize these findings, we reviewed the national community health policies from each of the six countries to determine the services that CHWs were expected to provide. In instances where policy documents were ambiguous, we also reviewed CHW training manuals for further clarity. We created a data extraction table to capture cadre name, selection process, responsibilities (for pregnant women, children under five years of age, everyone), training, and financial compensation. The key documents referenced included: National Strategic Plan for Community Health 2023–2027 in Guinea; National Community Health Strategic Plan 2021–2023 in Mali; Orientation and Reference Guide for Community Development Liaisons 2017 in Niger; National Strategic Plan Community Health Côte d’Ivoire 2022–2025 and Nutrition Intensification Days, Orientation Guide June 2024 in Cote d’Ivoire; National Strategic Plan for Community Health in DRC 2019–2022 and Training of Community Liaisons on Vitamin A Supplementation and Deworming January 2023 in the Democratic Republic of Congo; Community Health Policy 2020–2030 along with relevant modules from the Community Health Volunteer Basic Handbook in Kenya.

## Results

### Background characteristics

The study included 735 community health workers (CHWs) across six Sub-Saharan African countries: Guinea (*N* = 194), Mali (*N* = 138), Niger (*N* = 111), Côte d’Ivoire (*N* = 136), Democratic Republic of Congo (DRC) (*N* = 78), and Kenya (*N* = 78). The demographic characteristics of the CHWs varied across countries (Table 1). In four out of six countries, more than 40% of CHWs were female, with the highest proportion in Kenya (60%) and the lowest in Côte d’Ivoire (19%). A larger proportion of CHWs were over 30 years (recorded in three countries) with variation in the number of years of experience.

**Table 1:** Background characteristics of community health workers

	Guinea (*N* = 194)		Mali (*N* = 138)		Niger (*N* = 111)		Ivory Coast (*N* = 136)		DRC (*N* = 78)		Kenya (*N* = 78)	
	N	%	N	%	N	%	N	%	N	%	N	%
Sex												
Female	75	38.7	62	44.9	55	49.5	26	19.1	17	21.8	47	60.3
Age (years)												
<25	13	6.7	8	5.8	4	3.6	NA	NA	NA	NA	NA	NA
25–30	56	28.9	16	11.6	8	7.2	NA	NA	NA	NA	NA	NA
>30	125	64.4	114	82.6	99	89.2	NA	NA	NA	NA	NA	NA
Experience (years)												
<10	144	74.2	62	44.9	48	43.2	95	69.9	58	74.4	42	53.8
10–20	34	17.5	54	39.1	49	44.1	31	22.8	17	21.8	33	42.3
>20	16	8.2	22	15.9	14	12.6	10	7.4	3	3.8	3	3.8
Education												
None	4	2.1	10	7.2	8	7.2	1	0.7	1	1.3	17	21.8
Primary	10	5.2	47	34.1	40	36	23	16.9	9	11.5	13	16.7
Secondary1	69	35.6	40	29	59	53.2	58	42.6	17	21.8	43	55.1
Secondary2	86	44.3	30	21.7	1	0.9	41	30.1	44	56.4	5	6.4
University	25	12.9	11	8	3	2.7	13	9.6	7	9	NA	NA
Financial compensation												
Yes	139	71.6	104	75.4	75	67.6	114	83.8	60	77	NA	NA

NA, data for this specific intervention or variable was not available for the country due to lack of reporting; Secondary1, secondary 1 st cycle; Secondary2, secondary 2nd cycle

Most CHWs had some secondary education, though significant variations existed. In Mali and Niger, about one-third had only primary education, while a small percentage in Côte d’Ivoire and DRC reported having university-level education. A notable exception was Kenya, where 22% of CHWs had no formal education. In five out of six countries, more than 68% of CHWs reported receiving financial compensation for the services they provided. However, disparities were observed in the nature and consistency of this compensation across countries.

### CHW selection process, responsibilities, training, and compensation

In five out of six countries, CHWs were selected by their local communities (Table 2). However, the specifics of the selection process varied, with some providing more structured selection criteria than others. CHWs in all countries are expected to deliver a wide range of health and nutrition services, including antenatal care (ANC), growth monitoring and promotion (GMP), and management of wasting. However, there were notable differences in the scope of responsibilities, particularly regarding the distribution of preventive and curative commodities.

**Table 2:** CHW selection criteria, process, responsibilities, training, and compensation

	Guinea	Mali	Niger	Côte d’Ivoire	DRC	Kenya
Policy document	1) National Strategic Plan for Community Health 2023–2027	1) National Community Health Strategic Plan 2021–2023	1) Orientation and Reference Guide for Community Development Liaisons 2017	1) National Strategic Plan Community Health Côte d’Ivoire 2022–2025 2) Nutrition Intensification Days, Orientation Guide June 2024	1) National Strategic Plan for Community Health in DRC 2019–2022 2) Training of Community Liaisons on Vitamin A Supplementation and Deworming January 2023	1) Community Health Policy 2020–2030 2) Community Health Volunteer Basic Handbook (Modules 1–6, 9, 10)
Cadre name	Community Liaisons	Community Health Agents	Community Liaisons	Community Health Agents	Community Liaisons	Community Health Volunteers
Selection process	Individuals who live in the community and are selected by the community	Not mentioned	Selected by the community	Selected by the community	Individuals from the community selected by the community during an assembly organized by the village head	Individuals from the community selected at a community meeting called by the community leader or the community health committee
Responsibilities						
Pregnant women	Promote ANC, PNC, companionship during pregnancy; birth preparedness; importance of SBA during delivery; proper nutrition, and IFA Support birth delivery in the presence of SBA	Promote ANC, institutional delivery, proper nutrition	Promote ANC		Promote ANC, PNC SBA at delivery, follow-up and referral, IPT (and distribution)	Promote ANC, PNC, male involvement, development of birth plans, institutional delivery, family planning, proper nutrition, IFA, IPT
Children	Promote GMP; ECD; school attendance; birth registration; exclusive breastfeeding; complementary feeding, vaccination; newborn care including Kangaroo mother care; healthy eating Support Essential newborn care; VAS and deworming (distribute); screening; and referral for malnutrition Treat iCCM (malaria, diarrhea, pneumonia)	Promote GMP; ECD; birth registration; exclusive breastfeeding; nutritional advice; vaccination; Kangaroo mother care; VAS and deworming (distribute); sick child feeding; follow-up Support Home visits for newborns; use of chlorhexidine for newborns; campaigns [Vitamin A (distribute), polio, vaccines, deworming (distribute), use of mosquito nets], detection, monitoring, and referral of uncomplicated moderate and severe wasting Treat Uncomplicated malaria, diarrhea, ARI	Promote ENA/IYCF; screening for micronutrient deficiencies, malaria, and other tropical diseases; Support Management of wasting; distribute Vitamin A and deworming (distribute) Treat Childhood illness	Promote IYCF, screening and referral for wasting, vaccination, follow-up; VAS and deworming (distribute) Treat iCCM	Promote IYCF, GMP, ECD, vaccination; oral vaccines (distribute); VAS (distribute); deworming (distribute); breastfeeding; follow-up of newborns at home; referral Support Management of wasting (screening, referral, and treatment) Treat iCCM (diarrhea, malaria, pneumonia);	Promote GMP; ECD, newborn care, breastfeeding, vaccination, refer and follow-up, healthy diet; VAS (refer); deworming (distribute) Support Management of wasting (screening, referral, and treatment of moderate wasting) Treat iCCM (diarrhea, malaria, pneumonia), refer for micronutrient supplementation
All	Promote Reproductive health and family planning; use of mosquito nets; WASH; barrier measures (masks, social distancing) Support HIV/AIDS surveillance and care; surveillance of hepatitis, syphilis; referral to health center; detection and monitoring of non-communicable diseases; prevention of GBV Treat Dispense ARVs, DOTS for TB	Promote Reproductive health and family planning (distribute commodities); WASH (distribute aqua tabs); barrier measures (masks, social distancing); psychosocial care for women Support Surveillance and care for HIV/AIDs and other diseases	Promote Family planning, prevention of HIV	Promote Family planning (distribute commodities) Support Guinea worm surveillance and maintenance of water pumps; HIV/AIDs care Treat DOTS for TB	Promote Family planning, prevention of GBV, use of mosquito nets; WASH Support HIV/AIDs surveillance and care; people living with disabilities Treat DOTS for TB; NTDs (screening, referral, and treatment); NCD, screening, referral, and support)	Promote Family planning, healthy diet, WASH, NCD (screening and referral), mental health (screening and referral) Support Terminally ill care and support, disease surveillance; linking vulnerable households to social safety nets; support people living with disabilities Treat HIV/AIDS and TB
Training	Not mentioned	Not mentioned	No set curricula, standard manuals exist, but what training liaisons receive depends on NGOs	(1) Common training for all CHAs (15 days), (2) Specific training according to programs	1) Manual covering promotional aspects of role 2) Manuals for specific programs based on need	1) Basic modules 2) Technical modules based on need
Financial compensation	Yes	Yes	Yes (10,000 CFA per month; reimbursement of transportation costs, cost of attending meetings and trainings; sale of products)	Yes (20,000 CFA per month, bonus during campaigns, performance-based bonus, and payment during training)	No	Yes (stipend)

*ANC* antenatal care, *ARV* anti-retroviral, *DOTS* direct observed treatment short-course, *ECD* early childhood development, *ENA* essential nutrition actions, *GBV* gender-based violence, *GMP* growth monitoring and promotion, *iCCM* integrated community case management, *IFA* iron-folic acid, *IYCF* infant and young child feeding, *NCD* non-communicable diseases, *NTD* neglected tropical diseases, *PNC* post-natal care, *SBA* skilled birth attendant, *TB* tuberculosis, *WASH* water, sanitation, hygiene

Training for CHWs was inconsistently documented across countries. For example, Niger lacked a standardized training curriculum, relying instead on support from NGOs. In contrast, countries like Côte d’Ivoire and Kenya offered more structured training programs, with basic training provided to all CHWs and additional modules tailored to the specific needs of their geographic areas.

Financial compensation for CHWs was mentioned in policy documents in five out of six countries, though the nature and amount of compensation varied significantly. For instance, CHWs in Côte d’Ivoire and Niger received a monthly stipend, while those in the DRC did not receive any formal financial compensation.

### Provision of services to women

#### Iron and folic acid promotion and distribution

Among antenatal care (ANC) services, most CHWs (74–90%) reported promoting iron and folic acid supplements (IFA) among pregnant women (Table 3). However, a smaller proportion ranging from 4% in Kenya to 62% in DRC reported distributing IFA to pregnant women. This aligned with national policy documents that noted that the promotion of IFA was part of the CHW job description in four out of six countries, but none of the documents in the countries noted that CHWs should also dispense IFA. Three countries reported ANC counseling for CHWs, which ranged from 69% in Niger to 83% in Kenya.

**Table 3:**
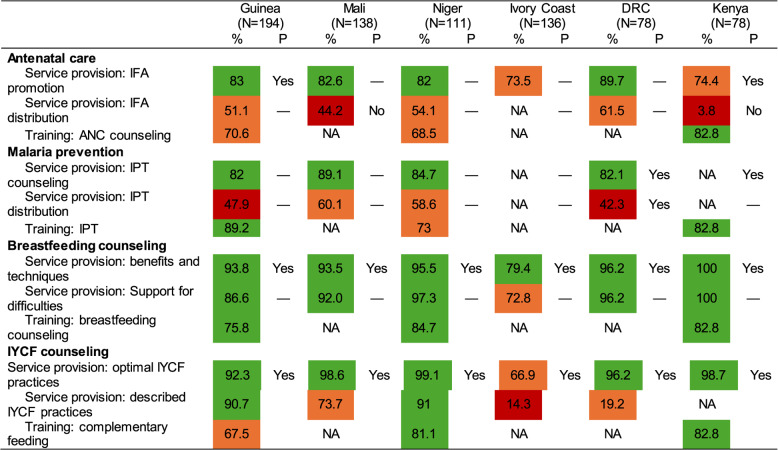
Services provided to women by community health workers

P, mentioned in policy documents

—, not mentioned in the documents reviewed

NA, data for this specific intervention or variable was not available for the country due to lack of reporting; IPT, intermittent preventive treatment

Legend: red < 50%, orange 50% to < 75%; green > = 75%

#### Malaria prevention

Most CHWs (82–89%) reported providing intermittent preventive therapy (IPT) for malaria prevention as part of ANC. However, a smaller proportion of CHWs reported distributing IPT, which ranged from 42% in DRC to 60% in Mali. Reported CHW training on IPT was moderately high (73–89%). However, other than the DRC and Kenya, very few of the policy documents mentioned that promotion of IPT was part of a CHW’s responsibility, reflecting inconsistencies between policy expectations and actual practice. Only the documents from DRC noted that the distribution of IPT medication was part of the CHW’s job description.

#### Breastfeeding counseling

Almost all CHWs noted that they counsel mothers on breastfeeding benefits and techniques, with reported service provision ranging from 79% in Côte d’Ivoire to 100% in Kenya. Similarly, a large proportion of CHWs reported supporting mothers with breastfeeding difficulties (73–100%). Not surprisingly, many CHWs mentioned that they had received breastfeeding counseling training (76–85%). While all policy documents mentioned that CHWs provide breastfeeding counseling, none had details on the support CHWs provided during breastfeeding difficulties (e.g., mastitis, engorged breasts, etc.).

#### Infant and young child feeding (IYCF) counseling

The majority of CHWs reported providing IYCF counseling, with high rates in Guinea (92%) and Niger (91%) and lower rates in Côte d’Ivoire (67%). When asked to describe the IYCF advice they shared with caregivers, the majority of CHWs in Guinea (91%), Niger (91%) and Mali (74%) were able to describe correct IYCF information. However, very few CHWs in Côte d’Ivoire (14%) and DRC (19%) were able to do so. While most CHWs in Kenya (83%) and Niger (81%) reported receiving training on complementary feeding, a smaller proportion did so in Guinea (68%).

### Provision of services to children under 5

#### Growth monitoring and promotion (GMP)

Except for Guinea (26%), most CHWs reported that they provided GMP (83–99%), though actual training in GMP ranged from a low of 55% in Guinea to a high of 83% in Kenya (Table 4). Policy documents in three countries explicitly included GMP as a CHW’s responsibility, though reported service provision did not always reflect this.

**Table 4:**
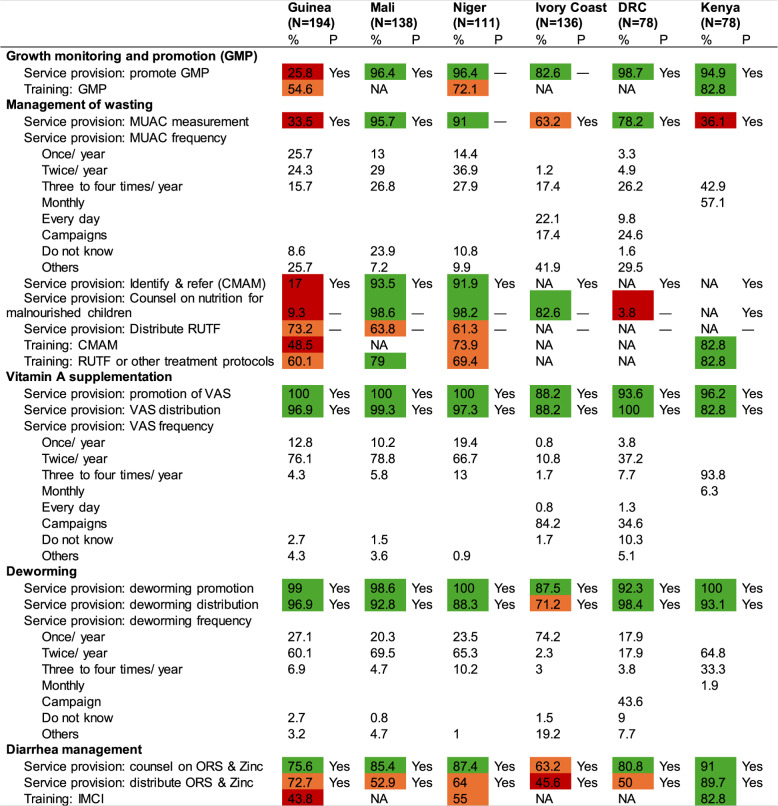
Services provided to children by community health workers

P, mentioned in policy documents

NA not applicable, data for this specific intervention or variable was not available for the country due to lack of reporting

—, not mentioned in the documents reviewed

Legend: red < 50%, orange 50% to < 75%; green > = 75%

#### Management of wasting

CHWs reported varied practices in screening for wasting using MUAC tape, ranging from a low of 34% in Guinea to a high of 96% in Mali. Reported screening frequencies also varied widely from monthly in Kenya (57%) to twice per year in Niger (37%), although over 40% of CHWs reported screening children for wasting using MUAC measurements monthly or three to four times per year. Very few CHWs noted taking MUAC measurements daily except in Côte d’Ivoire where 22% did so. In the DRC, MUAC screening during campaigns was most common (25%).

Identifying and referring children with wasting for treatment was high in Mali (94%) and Niger (92%), but low in Guinea (17%). A large proportion of CHWs in Mali, Niger, and Côte d’Ivoire (83–99%) noted counseling on nutrition for malnourished children while a lower proportion in Guinea and DRC (4–9%) did so. Distribution of RUTF was reported by 61–73% of CHWs in three countries. Training on community-based management of acute malnutrition (CMAM) or other treatment protocols was high in Kenya (83%) but low in Guinea (49–60%).

Among CHW responsibilities mentioned in policy documents, all noted identifying and referring children with wasting for CMAM, but only two mentioned counseling on nutrition for malnourished children, and none mentioned distribution of RUTF.

#### Vitamin A Supplementation (VAS) and deworming

Almost all CHWs reported promoting and distributing vitamin A supplements (VAS) and deworming tablets, aligning with national policies. However, the frequency of distribution varied, with most countries conducting distribution biannually, except for Kenya, where it occurred three to four times per year.

#### Diarrhea management

Counseling on the use of ORS and Zinc during diarrhea was high in Kenya (91%) but significantly lower in Côte d’Ivoire (63%). Similarly, the distribution of ORS and Zinc was high in Kenya (90%) but low in Côte d’Ivoire (56%), DRC (50%), and Mali (53%). Training on integrated management of childhood illnesses (IMCI), which covers ORS and Zinc, was highest in Kenya (83%) followed by Niger (55%), and Guinea (44%).

## Discussion

This study provides important insights into the compensation, service provision, and training of CHWs involved in delivering critical health and nutrition services in six Sub-Saharan African countries. We found that many CHWs received financial compensation, and their service provision largely focused on promotional and counseling activities, except for VAS and deworming tablets. Training levels varied with moderate to high coverage for services related to antenatal care (ANC), malaria prevention, and IYCF counseling. However, training for services directed at children, such as growth monitoring and promotion (GMP), community-based management of acute malnutrition (CMAM), and integrated management of childhood illnesses (IMCI), was moderate to high except in Guinea, where training on services for children was low. Importantly, we observed that national policy documents did not always clearly specify the services CHWs were expected to deliver, nor did they consistently align with the services that CHWs reported providing.

This study found that approximately two-thirds of the CHWs surveyed reported receiving financial compensation, a finding that in part aligns with the WHO guidelines advocating financial remuneration commensurate with CHWs’ scope of work and caution against reliance solely on performance-based incentives [17]. Evidence suggests that performance-based incentives may influence CHWs to focus more on tasks for which they receive financial incentives [18]. Policy documents from Côte d’Ivoire, Kenya, Niger, and Mali mention some form of financial compensation for CHWs. However, our study uncovered significant gaps, with 16–25% of CHWs in Côte d’Ivoire, Kenya, Niger, and Mali reporting that they did not receive the financial incentives specified in policy documents. This discrepancy highlights a critical area for policy implementation improvement.

CHWs can provide a variety of promotional, preventive, and curative services. In our study, most CHWs provided promotional services but were more likely to dispense preventive and curative commodities for children (e.g., VAS, deworming, ORS, and Zinc) than women (IFA, IPT). This may reflect how tasks are distributed among different members of the health workforce. CHW training correlated with their service provision. For example, in Guinea 49% of CHWs reported receiving CMAM training and only 17% reported screening and referring children with wasting for CMAM. In comparison, training and referral for CMAM were high in Mali. The same pattern was evident for IMCI training and ORS and Zinc distribution. This underscores the importance of adequately training CHWs to carry out their duties, a point consistently emphasized in previous studies [19].

The review of policy documents revealed inconsistencies and gaps in specifying the services CHWs are expected to provide. In several instances, even when a service was mentioned in the policy, the reported service provision was low, as seen with GMP and MUAC screening in Guinea, distribution of ORS and Zinc in Côte d’Ivoire and DRC, and MUAC screening in Kenya. Conversely, in three countries, more than 61% of CHWs reported that they distributed RUTF despite this role not being explicitly articulated in policy documents. This suggests that associated program documents or local adaptations might play a significant role in defining CHW responsibilities. It also indicates that CHWs may be mobilized for specific tasks in certain geographic areas based on local needs as previously noted [20]. Additionally, this suggests that CHWs’ roles may in part be dictated by the mandates and activities of non-governmental organizations (NGO) implementing activities in certain geographies, as reported by a previous study comparing service provision at health facilities and national procurement plans [15]. Such NGO-driven adaptations may fill service delivery gaps, but they can also contribute to inconsistency and confusion about CHW roles.

This study has several limitations. First, the findings on CHW service provision and training are based on self-reported data, which may not accurately reflect actual service delivery due to potential response bias. Second, the study focused on CHWs involved in VAS and deworming distribution, which may limit the generalizability of the findings to other community-level workers in the six countries. Specifically, the CHWs we interviewed might be more active or better trained than those not involved in the campaign, potentially leading to an overestimation of service provision and training levels. Additionally, the gender breakdown of health workers may differ from that of the general workforce. Third, the data reflect the experiences of CHWs in specific regions within each country and may not be representative of the entire country. Despite these limitations, these findings offer helpful insights into CHW compensation, training, and service provision, and highlight areas where improvements are needed to enhance the effectiveness of CHW programs in delivering essential health and nutrition services.

Our findings indicate that while many CHWs receive some form of financial compensation, gaps remain between what the policy provides for and policy implementation that could undermine their motivation and effectiveness. The strong correlation between training and service provision further emphasizes the importance of investing in comprehensive, context-specific training programs that equip CHWs with the necessary skills to carry out their roles effectively. Moreover, the inconsistencies between policy expectations and reported service delivery suggest that policy frameworks need to be more explicit and aligned with the realities on the ground. This alignment is crucial to ensure that CHWs are adequately supported and empowered to deliver a wide range of services, particularly those related to maternal and child nutrition, which are critical for long-term health and development.

To address these challenges, we offer recommendations for policy, practice, and further research. Policy: governments must harmonize CHW compensation structures, specifically, transitioning from stipends to salaries, and adopt tools like the CHW Assessment and Improvement Matrix to address implementation gaps. This tool includes assessments of worker motivation and recognition in addition to assessments of policy implementation regarding remuneration, supervision, and training [21]. Practice: programs should prioritize standardized training modules, such as Kenya’s structured curriculum, and integrate CHW feedback into service design and revision of policy documents. Research: the findings underscore the need to assess how policy revisions, such as formalizing RUTF distribution, impact service quality and equity. The success of these specific actions rests on the availability of adequate and sustained funding for CHW programs and the coordination of NGO partners who may support and fund CHWs only for specific activities [22]. By strengthening CHW programs, countries can improve the reach and quality of primary healthcare services, ultimately contributing to better health and nutrition outcomes for women and children in some of the world’s most vulnerable communities. These findings underscore the importance of aligning national policies with operational realities, including the contributions of NGO-supported programs, to ensure clarity, coherence, and sustainability of CHW roles.

## Conclusions

This study systematically quantifies the policy-practice gap across six countries, demonstrating how training adequacy and compensation models directly influence service provision. It uniquely highlights how CHWs often exceed policy mandates (e.g., RUTF distribution in three countries despite no policy mention) likely due to NGO-driven adaptations To address this, we offer several recommendations such as the need for governments to invest in CHWs, programs to prioritize CHW training structures, and future research to examine the impact of policy changes on service quality. These recommendations will ensure CHWs are equipped and empowered to play their critical role in the global health and nutrition landscape. They are, after all, the true unsung heroes of global health and nutrition.

## Supplementary Information

Supplementary Material 1.

## Data Availability

Data presented in the manuscript and analytic code will be made available upon reasonable request pending application and approval by the principal investigator.

## Abbreviations

ANC: Antenatal Care
CHW: Community Health Worker
CMAM: Community-Based Management of Acute Malnutrition
DRC: Democratic Republic of Congo
EA: Enumeration Areas
GMP: Growth Monitoring and Promotion
IFA: Iron Folic Acid
IMCI: Integrated Management of Childhood Illness
IPT: Intermittent Preventive Therapy
IYCF: Infant and Young Child Feeding
MUAC: Mid-Upper Arm Circumference
ORS: Oral Rehydration Solution
PEC: Post-Event Coverage
PPS: Probability Proportional to Size
VAS: Vitamin A Supplementation

## References

[1] WHO. Global strategy on human resources for health: Vision 2030. Geneva: WHO. 2016. Available from: https://iris.who.int/bitstream/handle/10665/250368/9789241511131-eng.pdf?sequence=1.

[2] CHIC. The Case for CHWs: Champions of the Health Systems. n.d. Available from: https://joinchic.org/resources/the-case-for-chws-champions-of-the-health-system/. Accessed 15 July 2024.

[3] LeBan K, Kok M, Perry HB. Community health workers at the dawn of a new era: 9. CHWs’ relationships with the health system and communities. Health Res Policy Syst. 2021;19(Suppl 3):116. 34641902 10.1186/s12961-021-00756-4 PMC8506091

[4] Glenton C, Javadi D, Perry HB. Community health workers at the dawn of a new era: 5. Roles and tasks. Health Res Policy Syst. 2021;19(Suppl 3):128. 34641903 10.1186/s12961-021-00748-4 PMC8506082

[5] VanderZanden A, Amberbir A, Sayinzoga F, Huda FA, Ntawukuriryayo JT, Mathewos K, et al. Evidence of health system resilience in primary health care for preventing under-five mortality in Rwanda and bangladesh: lessons from an implementation study during the millennium development goal period and the early period of COVID-19. J Glob Health. 2024;14:05023. 38963883 10.7189/jogh.14.05023 PMC11223753

[6] Kurian K, Lakiang T, Sinha RK, Kathuria N, Krishnan P, Mehra D, et al. Scoping review of intervention strategies for improving coverage and uptake of maternal nutrition services in Southeast Asia. Int J Environ Res Public Health. 2021;18(24):13292. 34948904 10.3390/ijerph182413292 PMC8701361

[7] Afsana K, Haque MR, Sobhan S, Shahin SA. BRAC’s experience in scaling-up MNP in Bangladesh. Asia Pac J Clin Nutr. 2014;23(3):377–84. 25164447 10.6133/apjcn.2014.23.3.22

[8] Garg S, Dewangan M, Patel K, Krishnendhu C, Nanda P. Role of Mitanin community health workers in improving complementary feeding practices under scaled-up home-based care of young children in a rural region of India. BMC Pediatr. 2023;23(1):171. 37046232 10.1186/s12887-023-03993-4 PMC10099942

[9] Janmohamed A, Sohani N, Lassi ZS, Bhutta ZA. The effects of community home visit and peer group nutrition intervention delivery platforms on nutrition outcomes in low and middle-income countries: a systematic review and meta-analysis. Nutrients. 2020;12(2):440. 10.3390/nu12020440. 10.3390/nu12020440 PMC7071285 32050577

[10] Janmohamed A, Doledec D, Dissieka R, Jalloh UH, Juneja S, Beye M, et al. Vitamin A supplementation coverage and associated factors for children aged 6 to 59 months in integrated and campaign-based delivery systems in four sub-Saharan African countries. BMC Public Health. 2024;24:1189. 38678255 10.1186/s12889-024-18707-3 PMC11055222

[11] López-Ejeda N, Charle Cuellar P, Vargas A, Guerrero S. Can community health workers manage uncomplicated severe acute malnutrition? A review of operational experiences in delivering severe acute malnutrition treatment through community health platforms. Matern Child Nutr. 2019;15(2):e12719. 30315743 10.1111/mcn.12719 PMC6587873

[12] Rahman M, Tariqujjaman M, Ahmed T, Sarma H. Effect of home visits by community health workers on complementary feeding practices among caregivers of children aged 6–23 months in 10 districts of Bangladesh. Front Public Health. 2022;10:1014281. 36777779 10.3389/fpubh.2022.1014281 PMC9912980

[13] Thorne-Lyman AL, Parajuli K, Paudyal N, Chitekwe S, Shrestha R, Manandhar DL, et al. To see, hear, and live: 25 years of the vitamin A programme in Nepal. Matern Child Nutr. 2022;18(1):e12954. 32108438 10.1111/mcn.12954 PMC8770656

[14] Njeru RW, Uddin MF, Zakayo SM, Sanga G, Charo A, Islam MA, et al. Strengthening the role of community health workers in supporting the recovery of ill, undernourished children post hospital discharge: qualitative insights from key stakeholders in Bangladesh and Kenya. BMC Health Serv Res. 2021;21(1):1234. 34775968 10.1186/s12913-021-07209-2 PMC8590969

[15] Dissieka R, Toure D, Singh A, Ndiath MM, Klemm RDW. Enhancing maternal and child health: a cross-national analysis of nutrition service readiness in Sub-Saharan Africa. Matern Child Nutr. In press. 10.1111/mcn.70064 42170946

[16] CHIC. ProCHW Policy Dashboard. Available from: https://joinchic.org/resources/prochw-policy-dashboard/. Accessed 15 July 2024.

[17] WHO. WHO Guideline on Health Policy and System Support to Optimize Community Health Worker Programmes. Geneva: World Health Organization; 2018. Available from: https://iris.who.int/bitstream/handle/10665/275474/9789241550369-eng.pdf. Accessed 15 July 2024. 30431747

[18] Kok MC, Broerse JEW, Theobald S, Ormel H, Dieleman M, Taegtmeyer M. Performance of community health workers: situating their intermediary position within complex adaptive health systems. Hum Resour Health. 2017;15(1):59. 28865471 10.1186/s12960-017-0234-z PMC5581474

[19] Schleiff MJ, Aitken I, Alam MA, Damtew ZA, Perry HB. Community health workers at the dawn of a new era: 6. Recruitment, training, and continuing education. Health Res Policy Syst. 2021;19(Suppl 3):113. 34641898 10.1186/s12961-021-00757-3 PMC8506097

[20] Hodgins S, Kok M, Musoke D, Lewin S, Crigler L, LeBan K, et al. Community health workers at the dawn of a new era: 1. Introduction: tensions confronting large-scale CHW programmes. Health Res Policy Syst. 2021;19(Suppl 3):109. 34641886 10.1186/s12961-021-00752-8 PMC8506102

[21] CHIC. Community Health Worker Assessment and Improvement Matrix (CHW AIM). Updated Program Functionality Matrix for Optimizing Community Health Programs. 2018. Available from: https://joinchic.org/resources/chw-aim/.

[22] Gichaga A, Masis L, Chandra A, Palazuelos D, Wakaba N. Mind the global community health funding gap. Glob Health Sci Pract. 2021;9(Suppl 1):S9–17. 33727316 10.9745/GHSP-D-20-00517 PMC7971370

