# Supplementary material for: Assessing the gap between policy and practice: community health workers’ contributions to nutrition services in Sub-Saharan Africa
Source: BMC Health Serv Res. 2025 Jul 1;25:838. doi: 10.1186/s12913-025-13044-6 (PMC12211232; doi:10.1186/s12913-025-13044-6)
Supplement: Supplementary file 1 — Supplementary Material 1. [file 12913_2025_13044_MOESM1_ESM.docx]

**Community Health Worker Questionnaire**

|  | **COVERAGE SURVEY** | 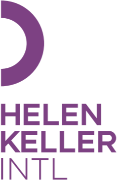 |
| --- | --- | --- |
| **Community Health Workers on Job Responsibilities Related to Nutrition Services** | | |

Information contained in this questionnaire is confidential. They are covered by statistical confidentiality and can only be published anonymously in accordance with [include regulation pertinent to each country].

**TO BE READ DURING THE SURVEY**

Good morning Madame / Sir,

My name is ______, We are conducting research on health services provided by your health center on vitamin A supplementation, antenatal and nutrition services for women and children. We would like to ask you some questions about these health services. The questions should only last for some time (15-20 minutes). Your advice will help plan health activities in your community for mothers and children.

You are free to choose to participate or not, you are also free to refuse to answer any of the questions. However, your opinion is very important in this study. Your answers will remain confidential. Do you want to participate?

Yes (continue) No END

**Q1 : GENERAL INFORMATION**

| **No.** | **QUESTIONS** | **ANSWERS** | **CODES** |
| --- | --- | --- | --- |
| **Q1.1** | Stratum |  | \|____\| |
| **Q1.2** | Health region |  | \|____\| |
| **Q1.3** | Health district |  | \|____\| |
| **Q1.4** | Ward |  | \|____\| |
| **Q1.5** | Health area / health center |  | \|____\| |
| **Q1.6** | Type of Health facility (CSR, CSU, …) |  | \|____\| |
| **Q1.7** | Zone | 1= 2= urban | \|____\| |
| **Q1.8** | Cluster no. |  | \|____\| |
| **Q1.9** | Surveyor code |  | \|____\|____\| |
| **Q1.10** | Team code |  | \|____\|____\| |
| **Q1.11** | Survey date | /______/______/______/  (day) (month) (year) | \|____\|____\|____\| |

**Q2: RESPONDENT PROFILE**

| **No.** | **QUESTIONS** | **ANWERS** | **CODES** |
| --- | --- | --- | --- |
| **Q2.1** | Gender | 1=Male  2=Female | \|____\| |
| **Q2.2** | Your level of education | 1= Any  2= Primary school  3= Secondary school 1st cycle  4= Secondary school 2^nd^ cycle  5= University | \|____\| |
| **Q2.3** | How long have you been CHW? | _____________ (in years) | \|____\| |
| **Q2.4** | Do you receive any monetary compensation for your services as a community health worker? | 1.Yes  2. No |  |
| **Q2.5** | If yes, how much did you receive? |  |  |

**Q3: ANTENATAL IRON AND FOLIC ACID SUPPLEMENTATION**

| **No.** | **QUESTIONS** | **ANSWERS** | **CODES** |
| --- | --- | --- | --- |
| **Q3.1** | Are you responsible for promoting the importance of antenatal iron and folic acid supplementation to pregnant women in your community? | 1.Yes  2. No |  |
| **Q3.2** | Do you distribute iron and folic acid supplements to pregnant women | 1.Yes  2. No |  |

**Q4: BREASTFEEDING COUNSELING**

| **No.** | **QUESTIONS** | **ANSWERS** | **CODES** |
| --- | --- | --- | --- |
| **Q4.1** | Do you provide counseling to mothers on the benefits and techniques of breastfeeding? | 1.Yes  2. No |  |
| **Q4.2** | Do you offer support and guidance to mothers who face difficulties with breastfeeding? | 1.Yes  2. No |  |

**Q5: INFANT AND YOUNG CHILD FEEDING**

| **No.** | **QUESTIONS** | **ANSWERS** | **CODES** |
| --- | --- | --- | --- |
| **Q5.1** | Do you provide counseling on optimal infant and young child feeding practices? | 1.Yes  2. No | \|____\| |
| **Q5.2** | How do you support mothers in practicing appropriate breastfeeding and complementary feeding | 1.Yes  2. No |  |

**Q6: GROWTH MONITORING, INCLUDING MEASURING MID-UPPER ARM CIRCUMFERENCE**

| **No.** | **QUESTIONS** | **ANSWERS** | **CODES** |
| --- | --- | --- | --- |
| **Q6.1** | Do you regularly measure the mid-upper arm circumference of children in your community? | 1.Yes  2. No | \|____\| |
| **Q6.2** | How often do you conduct these measurements in the last 6 months? | Once a month  Very 3 months  Every 6 months |  |
| **Q6.3** | Do you provide counseling to parents/caregivers on the significance of growth monitoring and interpreting the results? | 1.Yes  2. No |  |

**Q7: VITAMIN A SUPPLEMENTATION**

| **No.** | **QUESTIONS** | **ANSWERS** | **CODES** |
| --- | --- | --- | --- |
| **Q7.1** | Do you distribute vitamin A supplements to children in your community? | 1.Yes  2. No | \|____\| |
| **Q7.2** | How often do you provide these supplements to children? |  |  |
| **Q7.3** | Do you provide information to parents/caregivers about the importance and benefits of vitamin A supplementation? | 1.Yes  2. No |  |
| **Q7.4** | Do you have any equipment (BCC) that talks about Vitamin A? | 1= yes  2= No | \|____\| |

**Q8: PROVISION OF DEWORMING TO CHILDREN**

| **No.** | **QUESTIONS** | **ANSWERS** | **CODES** |
| --- | --- | --- | --- |
| **Q8.1** | Do you distribute deworming tablets to children in your community? | 1.Yes  2. No | \|____\| |
| **Q8.2** | How often do you provide these tablets to children? |  |  |
| **Q8.3** | Do you provide information to parents/caregivers about the importance and benefits of deworming? | 1.Yes  2. No |  |

**Q9: DIARRHEA TREATMENT**

| **No.** | **QUESTIONS** | **ANSWERS** | **CODES** |
| --- | --- | --- | --- |
| **Q9.1** | Do you educate parents/caregivers about appropriate home-based treatments for diarrhea in children? | 1.Yes  2. No | ~~\|____\|~~ |
| **Q9.2** | Do you distribute oral rehydration salts (ORS) and zinc tablets for diarrhea treatment? | 1.Yes  2. No |  |

**Q10: PROVISION OF IPT (INTERMITTENT PREVENTIVE TREATMENT) DURING PREGNANCY**

| **No.** | **QUESTIONS** | **ANSWERS** | **CODES** |
| --- | --- | --- | --- |
| **Q10.1** | Do you provide information on the importance of IPT during pregnancy to women in your community? | 1.Yes  2. No | \|____\| |
| **Q10.2** | Do you distribute IPT to pregnant women? | 1.Yes  2. No |  |

**Q11: TRAINING MODULES**

| **No.** | **QUESTIONS** | **ANSWERS** | **CODES** |
| --- | --- | --- | --- |
| **Q11.1** | Counseling during antenatal care visits? | 1.Yes  2. No | \|____\| |
| **Q11.2** | Nutritional assessment of pregnant women? | 1.Yes  2. No |  |
| **Q11.3** | Breastfeeding promotion? |  |  |
| **Q11.4** | Complementary feeding of young children? |  |  |
| **Q11.5** | Intermittent preventive treatment of malaria? |  |  |
| **Q11.6** | Community-based management of acute malnutrition? |  |  |
| **Q11.7** | Growth monitoring and promotion? |  |  |
| **Q11.8** | Integrated management of childhood illness? |  |  |
| **Q11.9** | Diagnosis and treatment of diarrhea? |  |  |
| **Q11.10** | Case management and treatment of malaria in children? |  |  |

|  | Save GPS coordinates |  |  |
| --- | --- | --- | --- |

***Thank you for your collaboration.***
